# Standardized Workflow and Analytical Validation of Cell-Free DNA Extraction for Liquid Biopsy Using a Magnetic Bead-Based Cartridge System

**DOI:** 10.3390/cells14141062

**Published:** 2025-07-11

**Authors:** Shivaprasad H. Sathyanarayana, Sarah B. Spracklin, Sophie J. Deharvengt, Donald C. Green, Margery D. Instasi, Torrey L. Gallagher, Parth S. Shah, Gregory J. Tsongalis

**Affiliations:** 1 The Laboratory for Clinical Genomics and Advanced Technology, Department of Pathology and Laboratory Medicine, Dartmouth Hitchcock Medical Center, Dartmouth Health, Lebanon, NH 03756, USA; 2 Dartmouth Cancer Center Phlebotomy, Department of Laboratory Services, Dartmouth Hitchcock Medical Center, Dartmouth Health, Lebanon, NH 03756, USA

**Keywords:** cell-free DNA, liquid biopsy, cfDNA extraction, TapeStation, reference materials, cancer detection

## Abstract

Circulating cell-free DNA (cfDNA) is an important biomarker for various cancer types, enabling a non-invasive testing approach. However, pre-analytical variables, including sample collection, tube type, processing conditions, and extraction methods, can significantly impact the yield, integrity, and overall quality of cfDNA. This study presents a comprehensive analytical validation of a magnetic bead-based, high-throughput cfDNA extraction system, with a focus on assessing its efficiency, reproducibility, and compatibility with downstream molecular applications. The validation was performed using a range of sample types: synthetic cfDNA spiked into DNA-free plasma, multi-analyte ctDNA plasma controls, Seraseq ctDNA reference material in a plasma-like matrix, extraction specificity controls, residual clinical specimen from patients, and samples from healthy individuals stored at room temperature or 4 °C for up to 48 h to assess stability. Extracted cfDNA was analyzed for concentration, percentage, and fragment size, using the Agilent TapeStation. Variant detection was evaluated using a next-generation sequencing (NGS) assay on the Seraseq ctDNA reference material. The results demonstrated high cfDNA recovery rates, consistent fragment size distribution (predominantly mononucleosomal and dinucleosomal), minimal genomic DNA (gDNA) contamination, and strong concordance between detected and expected variants in reference materials. The workflow also showed robust performance under different study parameters, variable sample conditions, including sample stability and integrity. Together, these findings confirm the efficiency and reliability of the evaluated cfDNA extraction system and underscore the importance of standardized pre-analytical workflows for the successful implementation of liquid biopsy for early cancer detection, therapeutic monitoring, and improved patient outcomes.

## 1. Introduction

Cell-free DNA (cfDNA) comprises fragmented DNA molecules released into the blood stream from various tissues and is detectable in multiple forms, including plasma, serum, urine, and other bodily fluids [1]. cfDNA is present in both healthy and diseased individuals, with most of the evidence suggesting that its release is primarily a consequence of apoptosis, though other physiological and pathological mechanisms contribute to its presence [2]. Apoptotic cells typically release shorter cfDNA fragments, while necrotic cells generate longer fragments [3]. Structurally, cfDNA consists of a nucleosome core wrapped by 147 base pairs (bp) of DNA, with linker DNA of approximately 20 bp, resulting in an average cfDNA fragment size of ~167 bp [4].

In cancer patients, a subset of cfDNA known as circulating tumor DNA (ctDNA) originates from malignant cells and is shed into the bloodstream. The detection of ctDNA has emerged as a valuable biomarker for early cancer diagnosis, tumor profiling, and disease monitoring [4]. To date, five liquid biopsy companion diagnostic assays have been approved by the U.S. Food and Drug Administration (FDA) for clinical utility. These assays help to determine patient eligibility for more than seventeen targeted therapies across several cancer types, including breast cancer, non-small-cell lung cancer (NSCLC), prostate cancer, colorectal cancer, and ovarian cancer, as well as one assay approved for use across all solid tumors [5,6]. In addition, genetic alterations identified in ctDNA such as somatic mutations, structural rearrangements, and epigenetic modifications enable insights into tumor origin, disease status, and the identification of potential novel cancer biomarkers [7]. Consequently, the process demands a highly sensitive and specific cfDNA extraction method followed by comprehensive molecular evaluation to ensure accurate and reproducible results [8].

Liquid biopsy, which involves the extraction and analysis of cfDNA from plasma, has emerged as a minimally invasive alternative to traditional tissue biopsies. This approach is increasingly utilized for cancer detection, recurrence prediction, and therapeutic resistance assessment [1,2,9]. Unlike tissue biopsies, which are limited by sampling bias and tumor heterogeneity, liquid biopsy offers a real-time, systemic view of tumor dynamics [10,11]. However, the analysis of cfDNA is technically challenging due to its low and variable abundance, high degree of fragmentation, and susceptibility to pre-analytical variability [12,13]. Additionally, factors such as pregnancy, strenuous exercise, underlying health conditions, and post-radiation cellular damage can influence cfDNA levels [14,15,16].

The efficiency of cfDNA extraction is contingent on several pre-analytical factors, including sample type, collection tubes, centrifugation parameters, storage conditions, extraction methodology, and downstream quantification approaches [17,18,19,20,21]. An ideal cfDNA extraction method should be rapid, robust, reproducible, and automatable to ensure high-quality yields suitable for downstream applications [22,23,24]. Various commercial extraction methods employ distinct extraction chemistries (Supplementary Table S1), including ethanol precipitation, silica membrane binding, and magnetic silica particle-based technologies [25]. Magnetic bead-based methods offer several advantages, including cost-effectiveness, speed, scalability, and compatibility with automation, often resulting in high-throughput processing and the extraction of high-quality cfDNA suitable for downstream applications [26]. The choice of an optimal extraction method depends on the intended downstream testing, required purity, and yield expectations.

Despite the growing clinical utility of cfDNA, technical challenges remain, particularly in achieving consistent and reliable extraction from plasma, where cfDNA concentrations can vary significantly depending on patient-specific pathological and physiological conditions [27]. Additionally, the isolation and quantification of cfDNA require workflows that are standardized, highly sensitive, and, most importantly, reproducible. The aim of this study was to develop and analytically validate a standardized, reproducible workflow for the extraction and quantification of cfDNA using a single-use cartridge-based, magnetic bead-assisted sample preparation system, with the goal of enabling reliable downstream molecular applications in clinical settings. Furthermore, by incorporating commercially available cfDNA/ctDNA reference materials, extraction-specific quality control reagents, and clinical specimens, we established a reliable and reproducible cfDNA extraction workflow designed to support both clinical and translational research applications.

## 2. Materials and Methods

### 2.1. cfDNA Extraction and Its Components

Pre-analytical variables influencing cfDNA extraction for liquid biopsy were systematically evaluated using commercially available reference standards, including the cfDNA reference standard (nRichDx, Irvine, California, USA), multi-analyte ctDNA plasma control (AcroMetrix, Thermoscientific, Benicia, CA, USA), and Seraseq ctDNA complete reference material (Seracare, Milford, MA, USA). Key parameters assessed in this study included extraction-specific quality control materials (Anchor Molecular, Pleasanton, CA, USA), sample stability, plasma separation, cfDNA extraction efficiency, quantification, and concentration range determination. These variables were optimized to enhance the reliability and reproducibility of cfDNA extraction, ensuring high-quality cfDNA suitability for downstream molecular applications such as next-generation sequencing, quantitative real-time PCR, or droplet digital PCR.

### 2.2. cfDNA Reference Standards

A commercially available cfDNA reference standard (nRichDX, Irvine, CA, USA) was employed for assay validation. The standard comprised five single-use tubes, each containing 1 mL of cfDNA at a concentration of 1 ng/µL in TE buffer. This reference material contained mononucleosomal DNA (mnDNA) harboring the *KRAS* p.G12V mutation, a well-established marker commonly used in spike-and-recovery experiments for cfDNA extraction studies. The reference material primarily consisted of mnDNA fragments of approximately 150 base pairs (bp), along with higher-order fragments including dinucleosomes (~340 bp) and trinucleosomes (~560 bp). To evaluate linearity, a range of starting sample volumes of 0.5 mL, 1 mL, 2 mL, 3 mL, 4 mL, 5 mL, and 6 mL were tested. DNA-free plasma (Zeptometrix, Franklin, MA, USA) was used as the matrix, and the cfDNA reference standard described above was spiked in at a concentration of 20 ng/mL. Similarly, varying input concentrations were generated by spiking different concentrations of 10 ng, 40 ng, 80 ng, 120 ng, 160 ng, and 200 ng of the nRichDx cfDNA reference standard material into 2 mL DNA-free plasma prior to extraction. Extraction recovery was further evaluated using quantitative real-time PCR (qPCR) targeting the *KRAS* p.G12V mutation. Primer and probe sequences are detailed in Supplementary Table S2. The qPCR reactions were carried out on the QuantStudio 3 Real-Time PCR System (ThermoFisher, Waltham, MA, USA) with the following thermal cycling conditions: initial denaturation at 95 °C for 10 min, followed by 40 cycles of denaturation at 95 °C for 15 s and annealing/extension at 60 °C for 1 min and 30 s. A standard curve was generated using a *KRAS* p.G12V reference standard (Horizon Discovery), and mutation quantification was performed based on the Cq values obtained.

### 2.3. Multi-Analyte ctDNA Plasma Control

AcroMetrix™ multi-analyte ctDNA plasma controls (Thermo Fisher Scientific, San Jose, CA, USA) were employed to evaluate assay performance. These controls included four variant allele frequency (VAF) levels: 0%, 0.1%, 0.5%, and 1%, enabling assessment across a range of mutation burdens. These controls consisted of fragmented synthetic DNA and genomic DNA from the GM24385 human cell line, suspended in a 2 mL normal human plasma matrix with a DNA concentration of 60 ng/mL. The control material includes a range of clinically relevant variants across multiple cancer types, comprising 7 single nucleotide variants (SNVs), 4 insertions/deletions (INDELs), and 2 copy number variations (CNVs). In this study, the reference material was utilized solely to assess cfDNA extraction efficiency, and no downstream molecular analyses were conducted.

### 2.4. ctDNA Complete Reference Material

The Seraseq ctDNA complete reference material (SeraCare, MA, USA), prepared in a 5 mL plasma-like matrix with a VAF of 0.1%, 0.5%, 1%, and 5% was included for the extraction evaluation. In addition, we also evaluated accuracy, precision, and reproducibility utilizing Seraseq ctDNA 0.5% complete reference material. This reference standard comprises 25 clinically relevant multiplexed variants across 16 genes, including 12 SNVs, 7 INDELs, 3 CNVs, and 3 structural variants (SVs).

### 2.5. Clinical Specimen Processing and Storage

A total of thirty-five residual clinical specimens (blood) were collected for this study. These samples had been previously submitted to a reference laboratory for routine ctDNA analysis. All specimens used in this study were processed within one to four hours of collection to ensure sample integrity. For the specificity assessment, blood samples were utilized from two healthy individuals using acid citrate dextrose (ACD) tubes. Additionally, to evaluate sample stability, 10 mL blood samples were used from four healthy individuals and aliquoted for storage under two conditions: two samples at room temperature (RT) and two samples at 4 °C for up to 48 h.

### 2.6. Extraction Specificity Controls

To ensure extraction quality control, cfDNA extraction specificity controls (Anchor Molecular, CA, USA) were employed. These included four distinct types: (1) high molecular weight DNA mixed with 170 bp fragments, (2) high-molecular-weight gDNA, (3) 170 bp fragments, and (4) negative control plasma. These controls were used to evaluate the specificity and efficiency of cfDNA extraction.

### 2.7. cfDNA Extraction and Quantification

**Figure 1:**
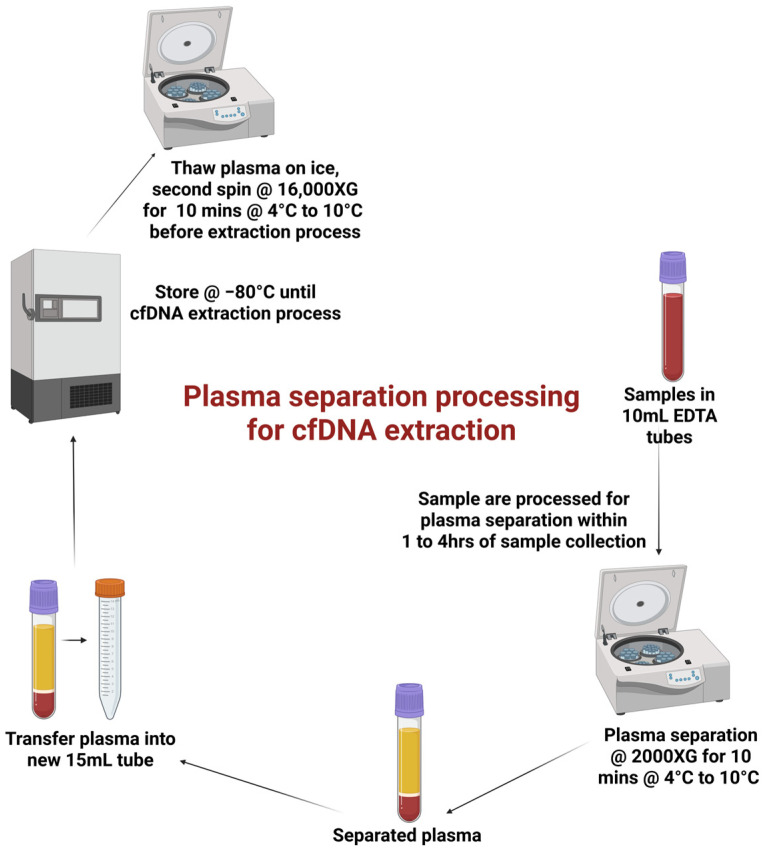
Extraction workflow—optimized plasma separation workflow for cfDNA extraction.

Plasma separation was performed according to the manufacturer’s standard operating procedure (nRichDX, CA, USA). Blood samples underwent an initial centrifugation at 2000× *g* for 10 min at 4 °C to 10 °C. The resulting plasma was carefully transferred into new storage tubes and stored at −80 °C until cfDNA extraction. Prior to extraction, plasma samples were thawed on ice and subjected to a second centrifugation at 16,000× *g* for 10 min at 4 °C to remove residual cellular debris (Figure 1).

cfDNA was extracted using the Revolution cfDNA Max 20 Kit (nRichDX, Irvine, CA, USA) in conjunction with the Revolution semi-automated extraction system, following the manufacturer’s instructions. The extraction workflow comprises five distinct steps: protease treatment, magnetic bead binding, bead capture, bead washing, and cfDNA elution. The extraction system used in this study accommodates plasma input volumes ranging from 1 mL to 50 mL. Following extraction, the final elution volume was at 50 µL, and cfDNA was quantified using the Cell-Free DNA ScreenTape Assay (Agilent Technologies, Santa Clara, CA, USA), which enabled assessment of cfDNA concentration, fragment size distribution, yield, purity, and residual gDNA contamination, thereby supporting downstream analytical validation.

## 3. Results

### 3.1. Evaluation of cfDNA Reference Standards

**Figure 2:**
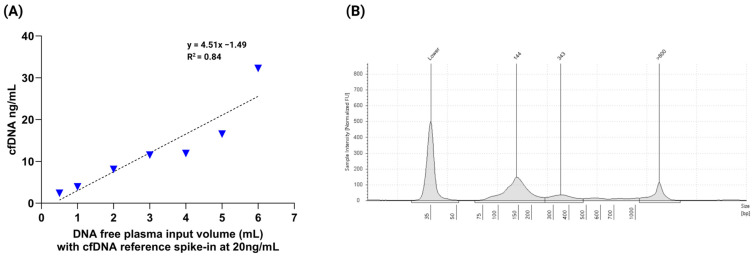
Linearity and fragment profile of cfDNA extraction using cfDNA reference material. (**A**) Linearity analysis of the cfDNA extraction method performed using nRichDX reference material spiked at a fixed concentration (20 ng/mL) into varying plasma input volumes (0.5–6 mL). A strong linear correlation (R^2^ ≥ 0.84) was observed between input volume and recovered cfDNA concentration, indicating consistent extraction efficiency across sample volumes. (**B**) Representative Agilent TapeStation electropherogram of the extracted cfDNA, displaying distinct at ~144 bp (mononucleosomal) and ~343 bp (dinucleosomal), with minimal gDNA traces observed at fragment sizes > 800 bp.

To assess the linearity and recovery of the cfDNA extraction method, plasma input volumes ranging from 0.5 mL to 6 mL were tested. DNA-free plasma samples were spiked with the nRichDX cfDNA reference standard at a concentration of 20 ng/mL. Recovered cfDNA concentrations, quantified using the TapeStation system (Agilent Technologies, Santa Clara, CA, USA), ranged from 2.37 ng/mL to 32.25 ng/mL, with an average concentration of 12.3 ± 9.29 ng/mL. The mean cfDNA percent was 73.1 ± 2.52, and the mean cfDNA fragment size was 231.6 ± 22.7 bp. Complete recovery data for each input volume is provided in the Supplementary Table S3. The study demonstrated a strong linear relationship between plasma input volume and recovered cfDNA concentration, with a coefficient of determination (R^2^) ≥ 0.84 (Figure 2A). A representative TapeStation electropherogram confirmed the cfDNA fragment profile, showing mononucleosomal (144 bp) and dinucleosomal (343 bp) cfDNA fragments (Figure 2B).

**Figure 3:**
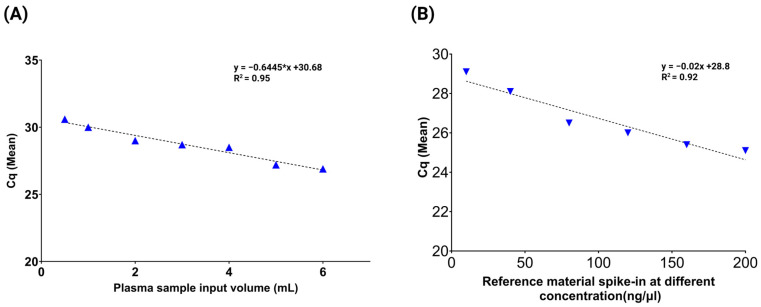
cfDNA reference material extraction recovery using qPCR targeting the *KRAS* p.G12V variant. (**A**) qPCR analysis of cfDNA extracted from plasma samples spiked with 20 ng/mL of *KRAS* p.G12V reference material across varying plasma input volumes. The results demonstrated efficient and consistent cfDNA recovery with a strong linear correlation (R^2^ ≥ 0.95), indicating volume-independent extraction performance. (**B**) cfDNA recovery efficiency was further assessed using a range of cfDNA spike-in concentrations. The qPCR data showed a robust linear relationship (R^2^ ≥ 0.92), confirming the quantitative accuracy and reliability of the extraction method across a dynamic input range.

To further assess recovery efficiency, a downstream qPCR assay targeting the *KRAS* p.G12V variant was performed. The qPCR results showed high recovery efficiency with a strong correlation (R^2^) ≥ 0.95 (Figure 3A). The mean Cq value was 28.7 ± 1.3 (range: 26.9–30.6), and the average cfDNA recovery was 76%, with a range of 43% to 95% across all extracted samples.

**Figure 4:**
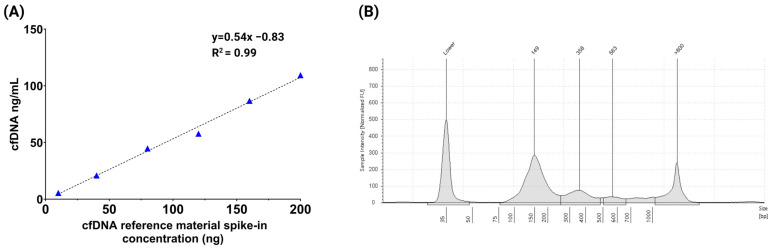
Data representing varying input concentrations. (**A**) Linearity assessment using the cfDNA reference material spiked into 2 mL of plasma at input concentrations ranging from 10 ng to 200 ng. The extraction demonstrated excellent linearity and high recovery efficiency with a strong correlation (R^2^ ≥ 0.99) using the Revolution workflow. (**B**) Representative TapeStation electropherogram showing cfDNA fragment size distribution, with distinct peaks at approximately149 bp (mononucleosomal), 358 bp (dinucleosomal), and 563 bp (trinucleosomal).

To further evaluate extraction performance, we assessed recovery across a range of input concentrations using the nRichDX cfDNA reference standard. Defined amounts of cfDNA 10 ng, 40 ng, 80 ng, 120 ng, 160 ng, and 200 ng were spiked into 2 mL of DNA-free plasma and recovered cfDNA concentrations were quantified using the TapeStation system. Recovered cfDNA concentrations ranged from 5.47 ng/mL to 109.3 ng/mL, with a mean concentration of 54.17 ± 35.7 ng/mL. The mean cfDNA percentage was 74.9 ± 0.4, and the mean fragment size was 230.9 ± 12.3 bp. Detailed recovery data for all concentrations are provided in Supplementary Table S4. The method demonstrated excellent linearity across input levels with a coefficient of determination (R^2^) ≥ 0.99 (Figure 4A). A representative TapeStation electropherogram showing cfDNA fragment profiles confirmed the presence of mononucleosomal (149 bp), dinucleosomal (358 bp), and trinucleosomal (563 bp) cfDNA fragments, as shown in Figure 4B.

In parallel, cfDNA recovery efficiency was evaluated using a qPCR assay targeting the *KRAS* p.G12V variant. The qPCR results demonstrated high extraction efficiency with a strong correlation (R^2^) ≥ 0.92 (Figure 3B). The mean quantification cycle (Cq) value was 26.6 ± 1.4 (range: 25.1–29.1), and the average recovery was 87% ranging from 75% to 100% across the tested concentrations.

### 3.2. Assessment of Multi-Analyte ctDNA Plasma Control

**Figure 5:**
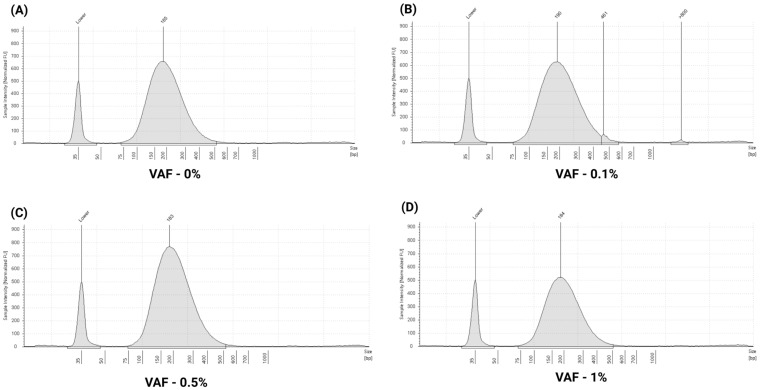
Representative TapeStation electropherogram profiles of multi-analyte ctDNA plasma control: electropherogram peaks illustrating cfDNA fragment size distributions across different VAF: (**A**) 185 bp (VAF—0%), (**B**) 190 bp (VAF—0.1%), (**C**) 183 bp (VAF—0.5%), and (**D**) 184 bp (VAF—1%). All profiles show well-defined cfDNA peaks with no gDNA contamination, as indicated by the absence or minimal signal above 800 bp.

Next, we evaluated extraction performance using 2 mL of multi-analyte ctDNA plasma control (60 ng/mL DNA concentration). Recovered cfDNA concentrations were measured using the Agilent TapeStation system. Across the four VAF levels, recovered cfDNA concentrations ranged from 38.7 ng/mL to 49.7 ng/mL, with a mean concentration of 49.2 ± 6.80 ng/mL. The mean cfDNA percentage was 98.3 ± 0.42, the mean fragment size was 211.5 ± 3.20 bp, and the average recovery was 82 ± 11.3%. Detailed recovery metrics are provided in Supplementary Table S5. Representative TapeStation electropherogram illustrating cfDNA fragment profiles is shown in Figure 5A–D.

### 3.3. Performance Evaluation of ctDNA Complete Reference Material

**Figure 6:**
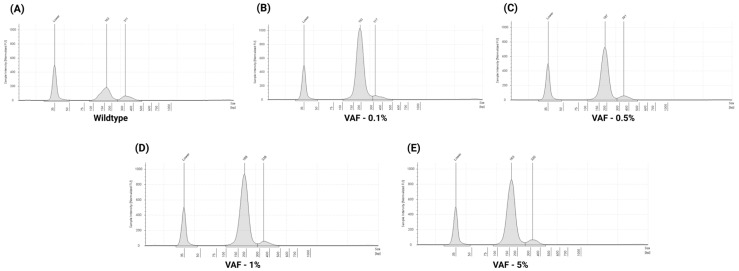
Representative TapeStation electropherogram profiles of ctDNA complete reference material: electropherogram peaks illustrating cfDNA fragment size distributions across different VAF: (**A**) 185 bp—mononucleosomal, 311—dinucleosomal (Wildtype), (**B**) 193 bp—mononucleosomal, 317—dinucleosomal (VAF—0.1%), (**C**) 187 bp—monomer, 341—dinucleosomal (VAF—0.5%), (**D**) 189 bp—mononucleosomal, 338—dinucleosomal (VAF—1%), and (**E**) 163 bp—mononucleosomal, 320—dinucleosomal (VAF—5%). Notably, no gDNA contamination was observed, as indicated by the absence of high-intensity peaks above 800 bp.

In this study, we also assessed the extraction performance using 5 mL of SeraCare ctDNA complete reference material (25 ng/mL DNA concentration). Recovered cfDNA concentrations were quantified using the Agilent TapeStation system. Across five different VAF levels, cfDNA concentrations ranged from 8.05 ng/mL to 25 ng/mL, and the mean concentration was 21.69 ± 7.39 ng/mL. The mean cfDNA percentage was 97.2 ± 1.46, the mean fragment size was 197.4 ± 9.85 bp, and the average cfDNA recovery was 86.7 ± 29.5%. Detailed recovery metrics are available in Supplementary Table S6. Representative TapeStation electropherogram illustrating cfDNA fragment profiles is shown in Figure 6A–E.

### 3.4. Precision and Reproducibility

**Figure 7:**
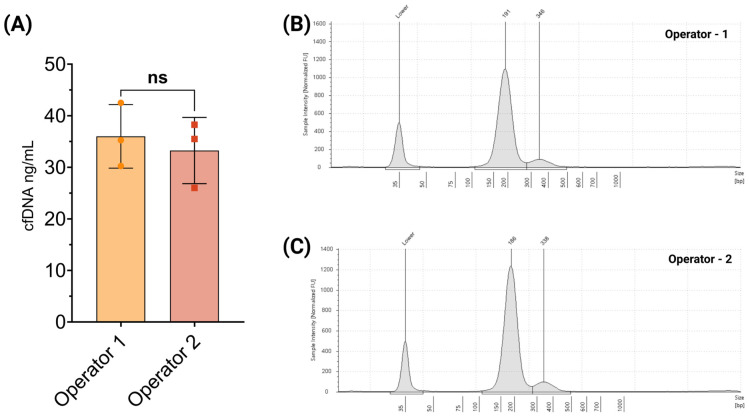
Precision and reproducibility of cfDNA extraction. (**A**) Total cfDNA yield obtained by two independent operators using the same extraction protocol and sample input, demonstrating consistent recovery and reproducibility. (**B**,**C**) Representative TapeStation electropherograms from each operator show highly similar cfDNA fragment profiles, with dominant peaks at ~180–190 bp (mononucleosomal) and ~340 bp (dinucleosomal), indicating reproducible fragment recovery and minimal inter-operator variability.

To evaluate the precision and reproducibility of the cfDNA extraction method, the Seraseq ctDNA complete reference material (0.5% VAF) was used in repeated extractions performed by two independent operators. For the intra-operator assessment, operator 1 conducted three independent extractions, yielding a mean cfDNA concentration of 36 ± 5.02 ng/mL (Figure 7A). In the inter-operator evaluation, operator 2 also performed three independent extractions, resulting in a mean cfDNA concentration of 33.2 ± 5.24 ng/mL (Figure 7A). The cfDNA percentage was comparable between operators, with operator 1 reporting a mean of 97.1 ± 0.28 and operator 2 reporting 97.9 ± 0.34. The mean cfDNA fragment size was 206.6 ± 1.88 bp for operator 1 and 199.3 ± 2.05 bp for operator 2. Although a minor difference in cfDNA yield was observed between operators, the consistency in cfDNA percentage and fragment size underscores the method’s robust reproducibility. Representative TapeStation electropherograms from operators 1 and 2 are shown in Figure 7B and Figure 7C, respectively.

### 3.5. Assessment of Specificity, Sample Storage Conditions, and Stability

**Figure 8:**
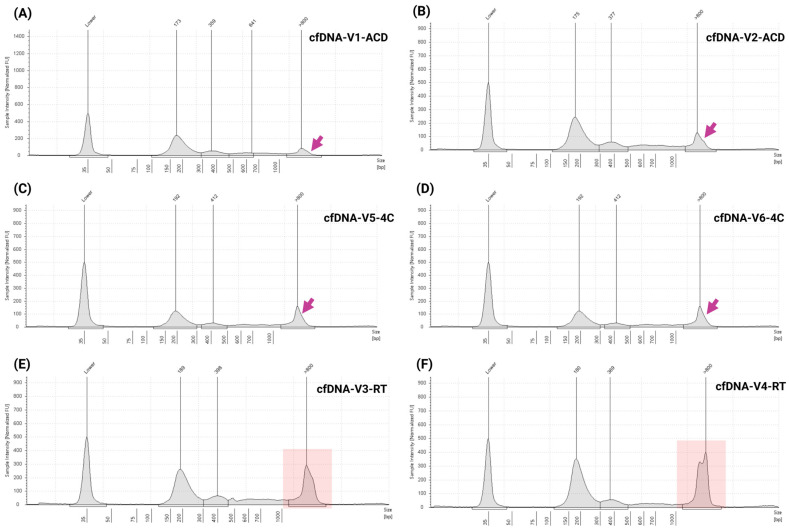
Impact of room temperature and 4°C storage on cfDNA stability in blood samples over a 48 h period compared to fresh samples. (**A**,**B**) TapeStation electropherograms of cfDNA extracted from freshly collected blood samples in ACD tubes, showing characteristic fragment peaks and minimal gDNA contamination. (**C**,**D**) Electropherograms from samples stored at 4 °C for up to 48 h demonstrating preserved cfDNA integrity with minimal gDNA presence compared to fresh samples. (**E**,**F**) Electropherograms from clinical samples stored at RT for up to 48 h, showing distinct cfDNA fragment peaks around ~180–190 bp (mononucleosomal), ~350–400 bp (dinucleosomal), along with significantly increased gDNA contamination (>800 bp). High-molecular-weight gDNA levels are indicated by arrows in panels (**A**–**D**), and highlighted in red boxes in panels (**E**,**F**), illustrating significant amount of gDNA due to the adverse impact of RT storage on cfDNA purity and integrity.

To evaluate analytical specificity, cfDNA was extracted from plasma samples collected in ACD tubes (non-K2EDTA tubes) from two healthy volunteers. The recovered cfDNA concentrations obtained were 5.57 ng/mL (cfDNA-V1-ACD) and 6.11 ng/mL (cfDNA-V2-ACD), with a mean concentration of 5.8 ± 0.3 ng/mL. The mean cfDNA percentage was 74.9 ± 2.7, and the mean fragment size was 275 ± 4.0 bp. TapeStation electropherograms confirmed distinct mononucleosomal and dinucleosomal peaks with minimal gDNA contamination as indicated by arrows (Figure 8A,B).

To assess the effect of storage temperature on cfDNA yield and integrity, blood samples from four healthy volunteers were collected in K2EDTA tubes. Two samples were stored at RT and two at 4 °C for up to 48 h prior to plasma separation and cfDNA extraction. Results demonstrated that cfDNA quality was affected by storage conditions. RT-stored samples yielded 2.59 ng/mL (cfDNA-V3-RT), and 8.34 ng/mL (cfDNA-V4-RT), with a mean cfDNA concentration of 5.47 ± 2.87 ng/mL, cfDNA percent of 56.6 ± 2.5, and a mean fragment size of 288.5 ± 4.5 bp. In contrast, samples stored at 4 °C yielded 5.90 ng/mL (cfDNA-V5-4C) and 6.76 ng/mL (cfDNA-V6-4C), with a mean concentration of 6.33 ± 0.42 ng/mL, a cfDNA percent of 58.8 ± 1.0%, and a mean fragment size of 275 ± 19 bp. Although one RT sample (cfDNA-V4-RT) showed a high yield, the other (cfDNA-V3-RT) demonstrated markedly lower recovery, highlighting variability under ambient conditions. TapeStation analysis corroborated these findings: 4 °C samples displayed clear mononucleosomal, and dinucleosomal peaks (Figure 8C,D), while RT samples exhibited signs of cfDNA degradation, low cfDNA percentage, and increased genomic DNA contamination (Figure 8E,F, highlighted in red boxes). These findings underscore the importance of processing samples when they are freshly collected or maintaining samples at 4 °C temporarily to preserve cfDNA integrity prior to processing.

**Figure 9:**
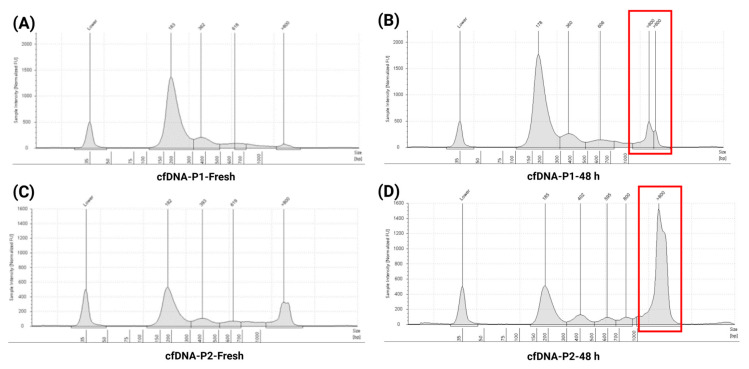
Assessment of cfDNA stability in blood samples stored at RT for up to 48 h compared to freshly processed samples. (**A**,**C**) TapeStation electropherograms of cfDNA extracted from freshly processed clinical blood samples, showing distinct cfDNA fragment peaks with minimal gDNA contamination. (**B**,**D**) Electropherograms of cfDNA extracted from matched clinical samples stored at RT for up to 48 h, demonstrating characteristic cfDNA fragment peaks around ~180 bp (mononucleosomal), ~370–380 bp (dinucleosomal), and ~600 bp (trinucleosomal), accompanied by marked gDNA contamination (>800 bp), as highlighted in the red boxes. These findings underscore the adverse impact of prolonged RT storage on cfDNA integrity and purity, and highlight the importance of timely plasma processing to minimize pre-analytical variability.

Using a similar approach, specificity and potential interference were assessed with two clinical specimens collected from cancer patients. The blood samples were in duplicate, with one set processed immediately and the other stored at RT for up to 48 h before plasma separation. cfDNA was extracted from all samples and concentrations; cfDNA percentage and fragment size data are summarized in Supplementary Table S7. TapeStation analysis revealed the presence of mononucleosomal, dinucleosomal, and trinucleosomal cfDNA peaks in all samples (Figure 9A–D). However, prolonged RT storage led to noticeable gDNA contamination in two patient samples (Figure 9B,D, highlighted in red boxes), suggesting compromised cfDNA integrity under these conditions. Variability in cfDNA stability was observed, likely influenced by the ctDNA fraction and underlying clinical characteristics of the patient samples.

### 3.6. Accuracy Assessment Using ctDNA Reference Standards and Clinical Samples

Accuracy was evaluated using the Seraseq ctDNA complete reference material containing 0.5% VAF in a plasma-like matrix. cfDNA extracted from this material was analyzed with an in-house validated ctDNA-Seq NGS assay [28]. Detected variants were compared to the known variants provided by the Seraseq reference dataset. As shown in Table 1, all 19 variants comprising 12 SNVs and 7 Indels were accurately detected by the ctDNA-Seq assay resulting in 100% concordance, confirming the analytical accuracy and sensitivity.

**Figure 10:**
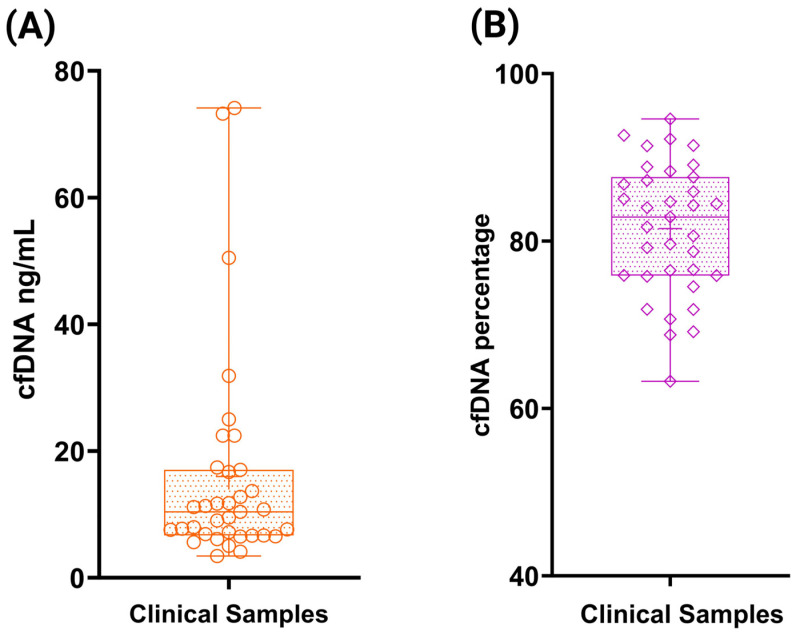
cfDNA extraction and quantification from clinical samples. (**A**) Concentration of extracted cfDNA from clinical samples quantified using the Agilent TapeStation and expressed in ng/mL. (**B**) Percentage of cfDNA in each patient sample as determined by TapeStation analysis.

For the final part, residual blood samples from 35 cancer patients were processed for plasma separation. Plasma volumes ranged from 2 mL to 6 mL in these samples, and cfDNA was subsequently extracted from each sample. TapeStation quantification revealed a concentration range from 3.43 ng/mL to 74.1 ng/mL, with a mean concentration of 15.9 ± 16.8 ng/mL (Figure 10A). The mean cfDNA percentage was 81.4 ± 7.72 (Figure 10B), and the mean fragment size was 266.1 ± 20.6 bp. Complete cfDNA concentration data for the clinical samples are provided in Supplementary Table S8.

### 3.7. Quality Control Assessment Using Extraction Specificity Controls

**Figure 11:**
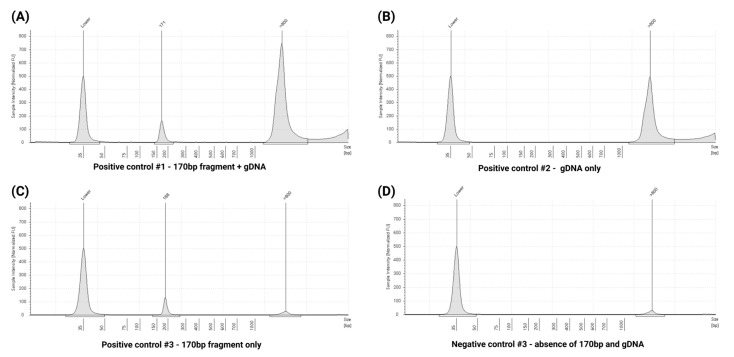
TapeStation electropherogram profiles of extraction specificity controls used for quality control assessments. (**A**) Positive control 1: Mixed sample containing both gDNA and 170 bp cfDNA fragments, demonstrating distinct peaks for each component. (**B**) Positive control 2: Sample containing gDNA only, showing a high-molecular-weight peak with no cfDNA signal. (**C**) Positive control 3: Sample containing 170 bp cfDNA fragments with minimal gDNA contamination, indicating successful cfDNA isolation. (**D**) Negative control: No detectable peaks, confirming the absence of contamination or non-specific amplification.

As a part of quality control, Anchor Molecular cfDNA extraction specificity controls were utilized. These served as positive controls, while DNA-free plasma without any spiked DNA was used as a negative control during cfDNA extraction. TapeStation quantification of the four extraction specificity controls yielded the expected results: positive control #1, containing both gDNA and 170 bp cfDNA, showed a peak at 171 bp alongside the presence of gDNA (Figure 11A). Positive control #2, consisting only of gDNA, exhibited a peak corresponding to gDNA with no cfDNA peaks (Figure 11B). Positive Control #3, composed solely of 170 bp cfDNA, demonstrated a peak at 188 bp with minimal gDNA contamination (Figure 11C). The negative control showed no relevant peaks, consistent with the absence of DNA (Figure 11D). These results confirm the specificity of the cfDNA extraction method and validate the quality control measures used in this study.

## 4. Discussion

Liquid biopsy is emerging as a transformative approach in clinical diagnostics and precision medicine, offering a non-invasive means to detect genetic variants that may be present in only a subset of cells. This capability is particularly advantageous in oncology, where early detection and monitoring of tumor-specific alterations can inform treatment decisions and improve patient outcomes [29,30,31,32,33,34]. The reliability of liquid biopsy assays is highly dependent on the consistency and efficiency of pre-analytical workflows, especially the extraction of cfDNA. Utilizing reference materials with well-characterized backgrounds and accurately defined spike-in variants is crucial for validating these assays and maintaining quality control across different laboratories. [35]. These reference materials enable the assessment of assay sensitivity, specificity, and reproducibility, ensuring consistency in cfDNA analysis across different settings. In this study, we systematically assessed the impact of various cfDNA reference materials on the performance of a magnetic bead-based, single-use cartridge sample preparation system. By employing well-characterized commercially available reference standards, including nRichDX cfDNA and Seracare ctDNA reference materials, we conducted a comprehensive evaluation of cfDNA under various conditions. This thorough analysis offers a more rigorous validation of the cfDNA extraction method compared to many previous reports [24,36,37,38,39,40].

Exogenous, nonhuman control materials have been developed to mimic cfDNA, featuring double-stranded structures and comparable fragment lengths [41,42]. These spike-in control DNAs are engineered to replicate the behavior of cfDNA while mitigating the contamination risk, making them suitable for clinical assay development and validation. However, cfDNA derived from mutant cell lines offers a more physiologically relevant model, as it closely resembles native cfDNA in terms of size distribution, blunt-ended fragmentation, and nucleosome positioning compared to synthetic oligonucleotides [43]. To minimize the impact of biological variability when evaluating cfDNA extraction methods, the incorporation of spike-in control DNA provides a standardized and reproducible approach for assessing recovery efficiency. In this study, we utilized nRichDX cfDNA reference standards and demonstrated strong linearity and robust analytical extraction sensitivity, with high-quality cfDNA concentrations. Complementary, downstream qPCR analysis confirmed cfDNA recovery rates of 75% to 85%, supporting the efficiency of the extraction process. Additionally, ctDNA extracted from the Seraseq ctDNA complete reference material (0.5% VAF) demonstrated strong concordance with expected results, capturing all 19 variants (12 SNVs and 7 Indels), achieving 100% concordance by ctDNA sequencing. These results highlight that spiking a defined quantity of exogenous cfDNA into plasma enables precise and consistent evaluation of extraction performance while reducing sample-to-sample variability.

Pre-analytical variables, including blood collection methods, storage duration, and centrifugation protocols, play a critical role in maintaining cfDNA integrity and have been extensively studied [44,45,46]. While recent guidelines for ctDNA mutation testing recommend collecting 2 × 10 mL of blood, commercially available liquid biopsy assays utilize plasma volumes ranging from 2 mL to 8 mL, and no universally accepted standard for blood collection volume has been established to date [16]. In our study, a single 10 mL blood sample was collected per patient, yielding mean plasma volumes ranging from 3 mL to 6 mL across clinical specimens.

Cell-stabilizing blood collection tubes have been shown to preserve ctDNA integrity when delays in plasma separation are unavoidable, allowing specimens to be stored at room temperature [17,47]. However, these specialized tubes, containing preservative agents, are approximately 50- to 100-fold more expensive than standard K2EDTA tubes. When rapid plasma processing is feasible, K2EDTA tubes are often preferred due to their cost-effectiveness, ease of use, and broad clinical availability [19]. In alignment with this practice, our study utilized 10 mL K2EDTA tubes for all blood collection. This approach is further supported by a retrospective analysis of 886 published cfDNA studies, which reported that 72% utilized K2EDTA tubes, underscoring their widespread adoption in liquid biopsy workflows [19].

Pre-processing storage conditions have a substantial impact on ctDNA integrity and concentration. Elevated or ambient temperatures can lead to leukocyte lysis, resulting in increased contamination from gDNA, particularly when using K2EDTA collection tubes. To mitigate this, most studies recommend plasma separation within two to four hours of sample collection [20]. Centrifugation step is another critical factor in cfDNA extraction. A single low-speed centrifugation step often leaves residual cellular components in plasma, increasing the risk of hemolysis and gDNA contamination, and is generally insufficient for obtaining true cell-free plasma [20]. In contrast, multiple studies have demonstrated that a two-step centrifugation protocol typically involving an initial low-speed spin followed by a high-speed spin, significantly reduces gDNA contamination and is therefore widely recommended for optimal cfDNA recovery [48,49,50].

In our study, blood samples collected in K2EDTA tubes were processed within or less than one hour of sample collection aligning with established pre-analytical best practices to preserve cfDNA integrity [19,20,21]. To assess sample stability, we also evaluated cfDNA quality from healthy volunteers and matched clinical samples stored at room temperature and 4 °C for up to 48 h. A primary limitation of this aspect of the study was a small sample size, and also, we were initially intended to collect 2 × 10 mL blood samples per subject for both fresh and 48 h delayed processing analyses, which could not be fully implemented. Nevertheless, our findings indicate that prolonged storage at room temperature adversely affects cfDNA quality and integrity, whereas storage at 4 °C better preserves cfDNA stability. Consistent with best practices and recommended guidelines [19,20], our study employed a two-step centrifugation protocol, with an initial centrifugation at 2000× *g* for 10 min at 4 °C, followed by a second centrifugation high spin at 16,000× *g* for 10 min at 4 °C. This approach effectively minimizes gDNA contamination and optimal cfDNA recovery. Following plasma processing separation, samples were stored at −80 °C until cfDNA extraction. To further maintain cfDNA integrity, plasma samples were thawed gradually on ice during the extraction process, minimizing the risk of degradation. Additionally, multiple freeze–thaw cycles were avoided to prevent cfDNA fragmentation and potential loss of low-abundance ctDNA.

Previous studies evaluating cfDNA extraction methods have primarily focused on comparing cfDNA yields across various kits and platforms, with an emphasis on maximizing cfDNA recovery efficiency [40,41,51,52,53]. However, ctDNA yield for molecular analysis is not solely dependent on plasma volume but also by individual-specific factors such as inflammation, gender, physical activity, and underlying pathophysiological conditions [54,55]. In cancer patients, ctDNA concentration can range from undetectable to 1000 ng/mL of plasma, with a mean concentration of approximately 10 ng/mL. Therefore, collecting 8–10 mL of plasma from 2 × 10 mL blood tubes typically provides sufficient cfDNA for most analytical applications [49,56,57].

In our study, we utilized a magnetic bead-based cfDNA extraction method capable of accommodating high plasma volumes (1–50 mL), offering flexibility and scalability across a wide range of sample inputs. Plasma volumes from our clinical cohort ranged from 2 to 6 mL, with a mean cfDNA concentration of 15.9 ± 16.8 ng/mL and a mean cfDNA percentage of 81.4 ± 7.72, determined by the Agilent TapeStation. Although the need for standardized cfDNA quality control guidelines has only recently gained attention [2], our study implemented both extraction specificity controls and well-characterized cfDNA reference materials to ensure rigorous method validation and reproducibility. Numerous reports have demonstrated that inconsistencies in pre-analytical conditions can significantly influence total cfDNA yield and the recovery from the plasma samples [44,58,59]. For clinical implementation, standardized protocols and workflows must be developed and validated through multi-center evaluation studies to establish consensus guidelines. These studies are essential to assess the impact of inter-laboratory procedure and its variability on ctDNA detection outcomes. Within the framework of external quality assessment (EQA), pre-analytical variables such as sample collection, handling, processing, and extraction must be clearly defined to establish performance criteria for clinical assay integration. Emphasis should be placed on developing robust, harmonized recommendations, guidelines, and standard operating procedures (SOPs) to ensure the accuracy, reproducibility, and clinical reliability of cfDNA extraction and subsequent downstream testing.

Lastly, a limitation of our study is the lack of a direct comparison with alternative extraction methods or platforms. Additionally, NGS data from the patient cohort and few of the reference materials were not included, as such analyses were beyond the scope of this manuscript and are currently being prepared for publication separately. Despite these limitations, our findings underscore the robustness, reproducibility, and clinical utility of the high-throughput cfDNA extraction workflow, supporting its integration in both research and diagnostic applications.

## 5. Conclusions

This study provides a comprehensive pre-analytical validation of a magnetic bead-based, high-throughput cfDNA extraction method by systematically evaluating critical factors that influence cfDNA yield, integrity, stability, and suitability for downstream molecular applications. By incorporating well-characterized reference materials and optimized plasma processing workflows, we demonstrated high extraction efficiency, strong reproducibility, and excellent concordance with reference standards as expected. Our findings emphasize the significant impact of pre-analytical variables including blood collection, storage temperature, and plasma processing by minimizing gDNA contamination and preserving cfDNA quality. These considerations are particularly relevant for ctDNA analysis, where minor pre-analytical inconsistencies can substantially impact the assay sensitivity and accuracy. Although a direct comparison with alternative extraction platforms was not within the scope of this study, our findings offer a robust and scalable framework for cfDNA isolation, supporting both clinical utility and research applications in precision oncology and non-invasive diagnostics.

## Figures and Tables

**Table 1:** Evaluation of cfDNA extraction and variant detection by ctDNA-Seq. cfDNA was extracted from SeraSeq ctDNA complete reference materials containing a defined set of clinically relevant molecular alterations, including twelve SNVs and seven INDELs. Downstream molecular analysis using the in-house ctDNA-Seq NGS assay successfully identified all 19 expected variants, demonstrating 100% concordance with the reference dataset. These results confirm the high extraction efficiency and analytical sensitivity of the workflow for mutation detection in cfDNA.

Gene	COSMIC ID	Seracare-Listed HGVS-c	Seracare-Listed HGVS-p	Transcript	Alteration Type	ctDNA-Seq Result	ctDNA-Seq Observed HGVS-c	ctDNA-Seq Observed HGVS-p
*AKT1*	COSM33765	c.49G>A	p.E17K	NM_001382430.1	SNV	Detected	c.49G>A	p.E17K
*ALK*	COSM28055	c.3522C>A	p.F1174L	NM_004304.5	SNV	Detected	c.3522C>A	p.F1174L
*ALK*	COSM144250	c.3604G>A	p.G1202R	NM_004304.5	SNV	Detected	c.3604G>A	p.G1202R
*BRAF*	COSM476	c.1799T>A	p.V600E	NM_004333.6	SNV	Detected	c.1799T>A	p.V600E
*BRCA1*	COSM219054	c.1961del	p.K654fs	NM_007294.4	Deletion	Detected	c.1961del	p.K654Sfs*47
*BRCA2*	COSM1738241	c.7934del	p.R2645fs	NM_000059.4	Deletion	Detected	c.7934del	p.R2645Nfs*3
*EGFR*	COSM6223	c.2235_2249del	p.E746_A750del	NM_005228.5	Deletion	Detected	c.2100_2114del	p.E701_A705del
*EGFR*	COSM12370	c.2240_2257del	p.L747_P753delinsS	NM_005228.5	Deletion	Detected	c.2105_2122del	p.L702_p708delinsS
*EGFR*	COSM6256	c.2254_2277del	p.S752_1759del	NM_005228.5	Deletion	Detected	c.2254_2277del	p.S752_1759del
*EGFR*	COSM6240	c.2369C>T	p.T790M	NM_005228.5	SNV	Detected	c.2369C>T	p.T790M
*EGFR*	COSM6224	c.2573T>G	p.L858R	NM_005228.5	SNV	Detected	c.2573T>G	p.L858R
*ERBB2*	COSM20959	c.2313_2324dup	p.Y772_A775dup	NM_004448.4	Insertion	Detected	c.2223_2234dup	p.Y742_A745dup
*KIT*	COSM1314	c.2447A>T	p.D816V	NM_000222.3	SNV	Detected	c.2435A.T	p.D812V
*KRAS*	COSM554	c.183A>C	p.Q61H	NM_004985.5	SNV	Detected	c.183A>C	p.Q61H
*KRAS*	COSM516	c.34G>T	p.G12C	NM_004985.5	SNV	Detected	c.34G>T	p.G12C
*KRAS*	COSM521	c.35G>A	p.G12D	NM_004985.5	SNV	Detected	c.35G>A	p.G12D
*NRAS*	COSM584	c.182A>G	p.Q61R	NM_002524.5	SNV	Detected	c.182A>G	p.Q61R
*PIK3CA*	COSM775	c.3140A>G	p.H1047R	NM_006218.4	SNV	Detected	c.3140A>G	p.H1047R
*PIK3CA*	NA	c.3204_3205insA	p.*1069Mext*3	NM_006218.4	Insertion	Detected	c.3204_3205insA	p.*1069Mext*3

## Data Availability

Data are available and can be found in Supplementary Materials.

## Supplementary Materials

The following supporting information can be downloaded at: https://www.mdpi.com/article/10.3390/cells14141062/s1, Table S1: Technical comparison of commercially available cfDNA extraction kits. Table S2: Primer and probe sequences used for qPCR targeting the *KRAS* p.G12V variant. Table S3: Summary of cfDNA recovery from reference standards across varying plasma input volumes demonstrating consistent extraction efficiency using the magnetic bead-based workflow. Table S4: Summary of cfDNA recovery from reference standards spiked into plasma at varying concentrations. Table S5: Detailed cfDNA recovery data from the multi-analyte ctDNA Plasma Control across four VAF levels (0%, 0.1%, 0.5%, and 1%). Metrics include recovered cfDNA concentration, fragment size, cfDNA percentage, and recovery efficiency (%), as measured by the Agilent TapeStation system. Table S6: Detailed data on cfDNA recovery from the ctDNA Complete Reference Material across five VAF levels (Wildtype, 0.1%, 0.5%, 1%, and 5%). The table includes recovered cfDNA concentration, cfDNA percentage, and fragment size, as assessed using the Agilent TapeStation system. Table S7: Sample stability assessment showing cfDNA recovery data from patient-derived plasma samples. Parameters include plasma input volume, recovered cfDNA concentration (ng/mL), cfDNA percentage, and fragment size (bp), as determined by Agilent TapeStation analysis. Table S8: Detailed data on cfDNA recovery from clinical samples. Parameters include plasma input volume (mL), recovered cfDNA concentration (ng/mL), cfDNA percentage, and fragment size (bp), as measured by the Agilent TapeStation system.
