# Supplementary material for: Standardized Workflow and Analytical Validation of Cell-Free DNA Extraction for Liquid Biopsy Using a Magnetic Bead-Based Cartridge System
Source: Cells. 2025 Jul 11;14(14):1062. doi: 10.3390/cells14141062 (PMC12293894; doi:10.3390/cells14141062)
Supplement: Supplementary file 1 [file cells-14-01062-s001.zip › cells-3654630-supplementary.pdf]

Supplementary Table S1: Technical comparison of commercially available cfDNA extraction kits

Parameter	QIAamp Circulating Nucleic Acid Kit	Qiagen EZ1 ccfDNA Kit	MagMAX Cell-Free DNA Isolation Kit	Maxwell RSC ccfDNA Plasma Kit	Revolution cfDNA Max 20 Kit
**Manufacturer**	Qiagen	Qiagen	ThermoFisher	Promega	nRichDX
**Extraction Method**	Silica membrane spin column	Magnetic bead, automated	Magnetic bead	Magnetic bead	Magnetic bead
**Sample Input Volume Range**	up to 5 mL	up to 10mL	up to 10mL	up to 5 mL	up to 50 mL
**Elution Volume**	20–50 µL	40–70 µL	15 - 50 µL	50–80 µL	20–100 µL
**Automation Compatible**	Yes (QiaCube)	Yes (EZ1 Advanced XL)	Yes (KingFisher instruments)	Yes (Maxwell RSC instrument)	Yes (Revolution semi-automated system)
**Max Sample Throughput**	Up to 24 samples	Up to 14 samples	Up to 24 samples	Up to 16 samples	Up to 24 samples
**Quality Controls Included**	No	No	No	No	No

Supplementary Table S2: Primer and probe sequences used for quantitative PCR (qPCR) targeting the KRAS G12V variant.

KRAS Probe	MGB Probe	/56-FAM/CTGTATCGTCAAGGCACT/3MGBEc/
KRAS G12V	Forward Primer	AAACTTGTGGTAGTTGGAGCAGT
KRAS G12V	Reverse Primer	CATATTCGTCCACAAAATGATTCTG

Supplementary Table S3: Summary of cfDNA recovery from reference standards across varying plasma input volumes demonstrating consistent extraction efficiency using the magnetic bead-based workflow.

	**Concentration ng/mL**	**cfDNA percentage**	**cfDNA fragment**
**cfDNA Vol 1mL**	2.37	74.7%	200.5
**cfDNA Vol 2 mL**	3.90	74.6%	209
**cfDNA Vol 3 mL**	8.08	74.9%	221.5
**cfDNA Vol 4 mL**	11.5	73.5%	235.5
**cfDNA Vol 5 mL**	11.8	71.2%	247
**cfDNA Vol 6 mL**	16.5	67.8%	273.5
**cfDNA Vol 7 mL**	32.2	75.1%	236
**Mean ± SD**	**12.36 ± 9.29**	**73.16 ± 9.29**	**231.86 ± 9.29**

Supplementary Table S4: Summary of cfDNA recovery from reference standards spiked into plasma at varying concentrations.

	**Concentration ng/mL**	**cfDNA percentage**	**cfDNA fragment**
**cfDNA Con 10**	5.47	74.2%	204
**cfDNA Con 40**	20.9	74.8%	230.5
**cfDNA Con 80**	44.7	75.2%	236
**cfDNA Con 120**	57.7	75.1%	237.5
**cfDNA Con 160**	86.7	75.6%	239.5
**cfDNA Con 200**	109.3	74.7%	238
**Mean ± SD**	**54.1 ± 35.7**	**74.9 ± 0.44**	**230.9 ± 12.3**

Supplementary Table S5: Detailed cfDNA recovery data from the Multi-Analyte ctDNA Plasma Control across four variant allele frequency (VAF) levels (0%, 0.1%, 0.5%, and 1%). Metrics include recovered cfDNA concentration, fragment size, cfDNA percentage, and recovery efficiency (%), as measured by the Agilent TapeStation system.

	**Concentration ng/mL**	**cfDNA fragment (bp)**	**cfDNA percentage**	**cfDNA Recovery**
AcroMetrix A – 1%	38.7	208	98.1%	64.5%
AcroMetrix B – 0.5%	57.7	213	99.0%	96.1%
AcroMetrix D – 0.1%	50.7	216	97.9%	84.5%
AcroMetrix E – 0%	49.7	209	98.5%	82.8%
**Mean ± SD**	**49.2 ± 6.80**	**211.5 ± 3.20**	**98.3 ± 0.42**	**82 ± 11.3**

Supplementary Table S6: Detailed data on cfDNA recovery from the ctDNA Complete Reference Material across five variant allele frequency (VAF) levels (Wildtype, 0.1%, 0.5%, 1%, and 5%). The table includes recovered cfDNA concentration, cfDNA percentage, and fragment size, as assessed using the Agilent TapeStation system.

	**Concentration ng/mL**	**cfDNA percentage**	**cfDNA fragment**
**SeraSeq Wildtype**	8.05	94.5%	203
**SeraSeq - 0.1%**	29	96.9%	205
**SeraSeq - 0.5%**	20.1	98.2%	201
**SeraSeq - 1%**	26.2	98.3%	200
**SeraSeq - 5%**	25	98.2%	178
**Mean ± SD**	**21.6 ± 7.39**	**97.2 ± 1.46**	**197.4 ± 9.85**

Supplementary Table S7: Sample stability assessment showing cfDNA recovery data from patient-derived plasma samples. Parameters include plasma input volume, recovered cfDNA concentration (ng/mL), cfDNA percentage, and fragment size (bp), as determined by Agilent TapeStation analysis.

	**Plasma Volume(mL)**	**Concentration (ng/mL)**	**cfDNA (%)**	**cfDNA fragment(bp)**
**cfDNA-P01-Fresh**	4.5	30.5	91.4%	254
**cfDNA-P02-Fresh**	4.3	9.56	67.6%	278
**cfDNA- P01- RT**	4.9	20.6	81.1%	255
**cfDNA-P02-RT**	4	8.30	34.1%	290

Supplementary Table S8: Detailed data on cfDNA recovery from clinical samples. Parameters include plasma input volume (mL), recovered cfDNA concentration (ng/mL), cfDNA percentage, and fragment size (bp), as measured by the Agilent TapeStation system.

	**Plasma Volume (mL)**	**Concentration ng/mL**	**cfDNA percentage (%)**	**cfDNA Fragment size (bp)**
cfDNA 1	3.78	8.20	68.8	306
cfDNA 2	4	3.92	84.27	259
cfDNA 3	4	0.67	86.77	298
cfDNA 4	4.02	3.65	84.67	268
cfDNA 5	4.1	2.23	75.89	299
cfDNA 6	4.3	4.64	83.99	265
cfDNA 7	4	0.68	89.08	267
cfDNA 8	4.82	12.11	74.55	251
cfDNA 9	4.45	8.88	75.78	293
cfDNA 10	3.28	4.41	76.48	283
cfDNA 11	3.6	6.61	69.15	297
cfDNA 12	3.3	4.80	70.68	260
cfDNA 13	4.5	7.26	87.63	252
cfDNA 14	4.38	4.48	87.24	251
cfDNA 15	4.5	4.25	92.18	246
cfDNA 16	4.2	2.00	88.34	260
cfDNA 17	3.83	6.96	79.2	290
cfDNA 18	4	6.26	71.82	290
cfDNA 19	5	5.25	79.63	265
cfDNA 20	2.15	5.53	75.87	284
cfDNA 21	2.05	7.48	82.87	257
cfDNA 22	2	0.99	92.62	232
cfDNA 23	3.8	9.95	78.73	266
cfDNA 24	3.81	6.47	76.57	273
cfDNA 25	4.4	7.65	85.03	251
cfDNA 26	4.6	6.54	63.24	272
cfDNA 27	5	2.99	88.86	239
cfDNA 28	4	14.60	71.84	262
cfDNA 29	4.88	1.57	91.36	219
cfDNA 30	4.38	7.68	80.61	286
cfDNA 31	4.75	7.52	81.68	273
cfDNA 32	4.5	2.23	91.41	254
cfDNA 33	4.75	2.93	84.44	263
cfDNA 34	4.65	4.27	85.89	257
cfDNA 35	5	2.87	94.58	228
**Mean ± SD**	**4.07±0.76**	**5.38±3.11**	**81.4±7.72**	**266.1±20.6**
